# Dystrophic cardiomyopathy: role of the cardiac myofilaments

**DOI:** 10.3389/fphys.2023.1207658

**Published:** 2023-06-09

**Authors:** Thomas G. George, Laurin M. Hanft, Maike Krenz, Timothy L. Domeier, Kerry S. McDonald

**Affiliations:** Department of Medical Pharmacology and Physiology, Dalton Cardiovascular Research Center, University of Missouri-Columbia, Columbia, MO, United States

**Keywords:** myofibrillar power, Duchenne muscular dystrophy, length dependent activation, cardiac myocytes, mavacamten

## Abstract

Dystrophic cardiomyopathy arises from mutations in the dystrophin gene. Dystrophin forms part of the dystrophin glycoprotein complex and is postulated to act as a membrane stabilizer, protecting the sarcolemma from contraction-induced damage. Duchenne muscular dystrophy (DMD) is the most severe dystrophinopathy, caused by a total absence of dystrophin. Patients with DMD present with progressive skeletal muscle weakness and, because of treatment advances, a cardiac component of the disease (i.e., dystrophic cardiomyopathy) has been unmasked later in disease progression. The role that myofilaments play in dystrophic cardiomyopathy is largely unknown and, as such, this study aimed to address cardiac myofilament function in a mouse model of muscular dystrophy. To assess the effects of DMD on myofilament function, isolated permeabilized cardiomyocytes of wild-type (WT) littermates and Dmd^mdx-4cv^ mice were attached between a force transducer and motor and subjected to contractile assays. Maximal tension and rates of force development (indexed by the rate constant, *k*_*tr*_) were similar between WT and Dmd^mdx-4cv^ cardiac myocyte preparations. Interestingly, Dmd^mdx-4cv^ cardiac myocytes exhibited greater sarcomere length dependence of peak power output compared to WT myocyte preparations. These results suggest dystrophin mitigates length dependence of activation and, in the absence of dystrophin, augmented sarcomere length dependence of myocyte contractility may accelerate ventricular myocyte contraction-induced damage and contribute to dystrophic cardiomyopathy. Next, we assessed if mavacamten, a small molecule modulator of thick filament activation, would mitigate contractile properties observed in Dmd^mdx-4cv^ permeabilized cardiac myocyte preparations. Mavacamten decreased maximal tension and *k*_*tr*_ in both WT and Dmd^mdx-4cv^ cardiac myocytes, while also normalizing the length dependence of peak power between WT and Dmd^mdx-4cv^ cardiac myocyte preparations. These results highlight potential benefits of mavacamten (i.e., reduced contractility while maintaining exquisite sarcomere length dependence of power output) as a treatment for dystrophic cardiomyopathy associated with DMD.

## Introduction

Dystrophinopathies are a class of diseases that arise from mutations in the dystrophin gene. Dystrophin is a protein that connects the cytoskeleton of striated muscle cells to the extracellular matrix and forms part of the dystrophin associated glycoprotein complex (DGC). An absence of dystrophin typically presents as skeletal muscle weakness and is associated with increased susceptibility to contraction-induced damage. The most severe form of dystrophinopathy is Duchenne muscular dystrophy (DMD). DMD is a X-linked recessive disease caused by a mutation in the dystrophin gene, that, in most cases, leads to its absence in the DGC of striated muscle cells.

DMD affects 1 in 5,000 boys born in the United States (Nigro et al., 1990). Symptoms typically appear between the ages of 2–5 years, with patients presenting with a waddling gait and difficulty climbing stairs. From this, symptoms progressively worsen eventually resulting in frequent falls and a loss of ambulation, usually at ages 7–13 years. Symptoms continue to deteriorate with weakness progressing to the upper limbs. Historically, patient mortality results from respiratory deficits, most notably impairments of the diaphragm, in the late teens to early twenties. Advances in treatment of skeletal muscle have extended life span in DMD patients (Eagle et al., 2002; Ameen and Robson, 2010; Cheeran et al., 2017; Shih et al., 2020) but has unmasked a cardiac component of the disease, known as dystrophic cardiomyopathy. From dystrophic cardiomyopathy heart failure can develop, which in now a primary cause of mortality (Kogelman et al., 2018). As such, it is pertinent to elucidate the mechanisms by which DMD causes heart failure.

Dystrophin has been postulated to act as a membrane stabilizer in striated muscle. This idea is supported by studies that examined the effect of contraction on sarcolemmal stability in models of DMD. In the absence of dystrophin sarcolemma instability manifested as greater membrane damage in response to stress (Houang et al., 2018). Muscle membrane damage has been observed in the form of micro-tears, which leads to extracellular calcium influx, and, in turn, activates sarcoplasmic reticulum calcium release resulting in intracellular calcium overload that exacerbates muscle cell death (Houang et al., 2018). Consistent with this idea, isolated hearts from Dmd^mdx^ mice exhibited elevated lactate dehydrogenase (LDH) levels, a marker of cardiac injury, in response to afterload challenge, in addition, there was greater cardiac myocyte sarcolemma damage and necrosis in response to isoproterenol and aortic constriction in Dmd^mdx^ hearts *in vivo* (Danialou et al., 2001). Together, these implicate a cardioprotective role for dystrophin and reducing cardiac work may improve DMD patient outcomes.

It is postulated that DMD also could affect the contractile properties of cardiac myofilaments. Some studies have investigated cardiac muscle contractile properties using Dmd mouse models. Twitch force was decreased in isolated, intact trabeculae muscle of 2-month Dmd^mdx^ mice (Janssen et al., 2005). This previous study also utilized a more severe double knockout (DKO) mouse, which was lacking both dystrophin and utrophin, a protein postulated to compensate for the loss of dystrophin in Dmd^mdx^ mice (Blake et al., 1996; Janssen et al., 2005). In the DKO mice, a further reduction was observed in twitch force (Janssen et al., 2005). In another study, changes in contraction were seen before the presence of fibrosis or necrosis, promoting the idea that decreased function is directly due to a loss of dystrophin and not the consequence of cell death or fibrotic remodeling (Quinlan et al., 2004). Additionally, the cardiac relaxation times were significantly increased in Dmd^mdx^ mice at 12–14 weeks (Sapp et al., 1996), further indicating the effect a loss of dystrophin may have upon contractile function in cardiac muscle. To our knowledge, there are no studies that have investigated cardiac myofilament dynamic contractile properties including rates of force, loaded shortening and power output and their sarcomere length dependence in Dmd^mdx^ mice. Thus, we hypothesized that dynamic contractile properties and their length dependence would be depressed in cardiac myofilaments from Dmd^mdx-4cv^ mice.

## Methods

### Animal model

All procedures involving animals were performed in accordance with an approved protocol from the Animal Care and Use Committee of the University of Missouri. The Dmd^mdx-4cv^ mouse model was used for this study, as a model that approximates human DMD (Yucel et al., 2018). For instance, *Dmd* ^*mdx-4Cv*^ mice have 10 times fewer revertant fibers in skeletal muscle cross-sections, which could impact cardiac muscle cells (Danko et al., 1992; Pigozzo et al., 2013) and led to a slightly more severe and less variable cardiac phenotype in *Dmd* ^*mdx-4Cv*^ compared to the classic *Dmd* ^*mdx*^ mice. Mice were between the ages of 4–12 months. Wild-type (WT) littermate mice were used as the control group.

### Solutions

Relaxing solution for permeabilized cardiac myocytes contained: 1 mM DTT, 100 mM KCl, 10 mM Imidazole, 2.0 mM EGTA, 4.0 mM ATP, 1 mM (free, 5 total) MgCl_2_. Minimal Ca^2+^ activating solution (pCa 9.0) for experimental protocol contained: 7.00 mM EGTA, 20 mM Imidazole, 5.42 mM MgCl_2_, 72.37 mM KCl, 0.016 mM CaCl_2_, 14.50 mM PCr, 4.7 mM ATP. Maximal Ca^2+^ activating solution (pCa 4.5) for experimental protocol contained: 7.00 mM EGTA, 20 mM Imidazole, 5.26 mM MgCl_2_, 60.25 mM KCl, 7.01 mM CaCl_2_, 14.50 mM PCr, 4.81 mM ATP. A range of Ca^2+^ concentrations for experiments was prepared by varying combinations of minimal and maximal Ca^2+^ solutions. Pre-activating solution contained: 0.5 mM EGTA, 20 mM Imidazole, 5.42 mM MgCl_2_, 98.18 mM KCl, 0.016 mM CaCl_2_, 14.50 mM PCr, and 4.8 mM ATP. The submaximal calcium concentration was determined by selecting the pCa solution that elicited ∼50% of maximal force obtained in pCa 4.5. For mavacamten experiments, 0.5 µM mavacamten was added to all pCa solutions resulting in a reduction of maximal force by ∼50%, in agreement to previous studies (Awinda et al., 2020; Awinda et al., 2021; Sewanan et al., 2021). For experiments in the presence of 0.5 µM mavacamten, the submaximal calcium concentration was determined by selecting the pCa solution with mavacamten that elicited ∼50% maximal force obtained in pCa 4.5 containing mavacamten.

### Permeabilized cardiac myocyte preparations

Mice were weighed and then anaesthetized using isoflurane and their hearts were excised. Following excision, the heart was placed into an ice-cold relaxing solution. The left ventricle was cut into 2–3 mm pieces and then placed into a Waring blender for 5–10 s. The resulting suspension of cells was then centrifuged for 105 s at 165 g. Myocytes were then permeabilized using Triton X-100.

For immunostaining, a drop of permeabilized cardiac myocyte preparations were added to a cover slip, fixed in 4% paraformaldehyde, washed in phosphate buffered saline (PBS), and blocked in 3% bovine serum albumin (BSA) for 1 h. Next the myocyte preparations were incubated with primary antibody to dystrophin (MANDYS1 3B7, Developmental Studies Hybridoma Bank, Iowa City, IA) overnight at 4°C. Coverslips were washed three times in PBS, then incubated in ALEXA 488 anti-mouse secondary antibodies for 1 hour at 37°C. Coverslips were sealed and imaged via confocal microscopy.

For mechanical measurements, permeabilized myocytes were attached between a force transducer and torque motor by placing a myocyte preparation into a stainless steel-troughs (25 gauge) and secured by overlaying the ends of the myocyte with 0.5 mm length of 4–0 monofilament nylon sutures (Ethicon, Inc.), then the sutures were secured into the troughs utilizing loops of 10–0 monofilament (Ethicon, Inc.). Attachment took place using a stereomicroscope (90x zoom). The myocyte preparation apparatus was transferred and mounted onto an inverted microscope (model IX-70, Olympus Instrument Co., Japan). A capacitance-gauge transducer and 10x amplifier (Aurora Scientific, Inc, Aurora ON, Canada) was used for mechanical measurements. Changes in length were introduced using a DC torque motor (model 308, Aurora Scientific, Inc.) by voltage commands from a personal computer using a 16-bit D/A converter (AT-MIO-16E-1, National Instruments Corp., Austin, TX, United States). Length and force signals were digitized at 1 kHz and stored on a personal computer in LabView for Windows (National Instruments Corp.). The length of the sarcomere was measured using a IonOptix SarcLen system (IonOptix, Milton, MA), which uses a fast Fourier transform algorithm on the video image of the myocyte. Once attached, the relaxed cardiomyocyte preparation was adjusted to a SL of ∼2.25 µm and passive tension was assessed by slacking the preparation in pCa 9.0 solution. Force, rate of force, and loaded shortening velocities were made at 15°C ± 1°C at two sarcomere lengths (see Figure 2 for representation of experimental workflow) (Hanft et al., 2021). A final force measurement was made at long sarcomere length in pCa 4.5 solution, and if was found to be below 70% of initial maximal force, data from that myocyte were discarded.

### Data and statistical analysis

Force redevelopment following a slack-restretch maneuver was fit by a single exponential equation:

$$F=F_{\max}\left(1\;–\;e^{-ktrt}\right),$$

where F is force at time *t*, F_max_ is maximal force, and *k*_*tr*_ is the rate constant of force development.

Myocyte length traces were fit to a single decaying exponential equation:

$$L={Ae}^{-kt}+C$$

Where L is cell length at time t, A and C are constants with dimensions of length, and the rate constant of shortening is *k*. Velocity of shortening at time, *t*, was determined by the slope of the tangent to the fitted curve at *t*. Loaded shortening velocities were calculated from the onset of force clamp at *t* = 0 msec. The Hill equation (Hill, 1938) was used to fit hyperbolic force-velocity curves to relative force-velocity data.

$$\left(P+a\right)\;\left(V+b\right)=\left(P_{0}+a\right)\;b$$

P is the force during shortening at velocity V; P_0_ is the maximal isometric force; a is a constant with dimensions of force, while b is a constant with dimensions of velocity. Force-velocity data is normalized to isometric force. Force was multiplied by velocity to obtain power-load curves, and peak normalized power output (PNPO) values were obtained by the multiplication of relative force at optimum power by velocity at optimum power. Curves were fit using commercial software (SigmaPlot) and a custom program written using Qbasic. To calculate cross-sectional area, the cell shape was assumed to be elliptical so the width was multiplied by a factor of 0.7 on mnor axis (Strang et al., 1994).

WT and Dmd^mdx-4cv^ cardiac myocyte preparations were compared using Student’s *t* test. Within group sarcomere length dependence of *k*_*tr*_ values, force-velocity and power-load curve parameters were compared using paired *t* tests. WT and Dmd^mdx-4cv^ preparations were compared before and after mavacamten treatment using a paired *t*-test. Between group differences in response to mavacamten were compared using either a Mann-Whitney test (Figure 9B) or One-Way ANOVA (Figure 9C). A *p*-value below 0.05 was considered significant. Significance was denoted by *. N = number of mice/group and all data shown are biological replicates.

## Results

**FIGURE 1:**
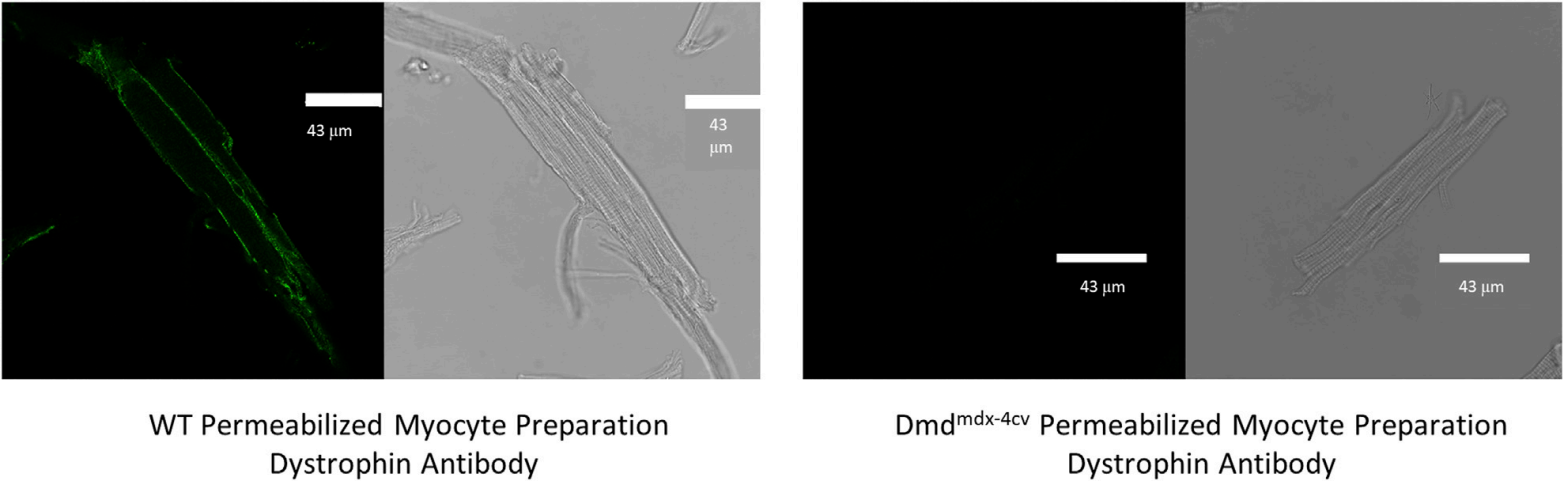
Immunofluorescent images show the presence and absence of dystrophin staining in permeabilized cardiac myocytes from wildtype (WT) and Dmd^mdx-4cv^ mouse hearts, respectively.

Immunofluorescent staining was performed to assess dystrophin content in permeabilized cardiac muscle suspensions after mechanical disruption of wildtype (WT) and Dmd^mdx-4cv^ hearts. While dystrophin remained present in permeabilized cardiac muscle suspensions from WT hearts, dystrophin was absent in permeabilized muscle preparations from Dmd^mdx-4cv^ hearts (Figure 1).

### Contractile properties

**FIGURE 2:**
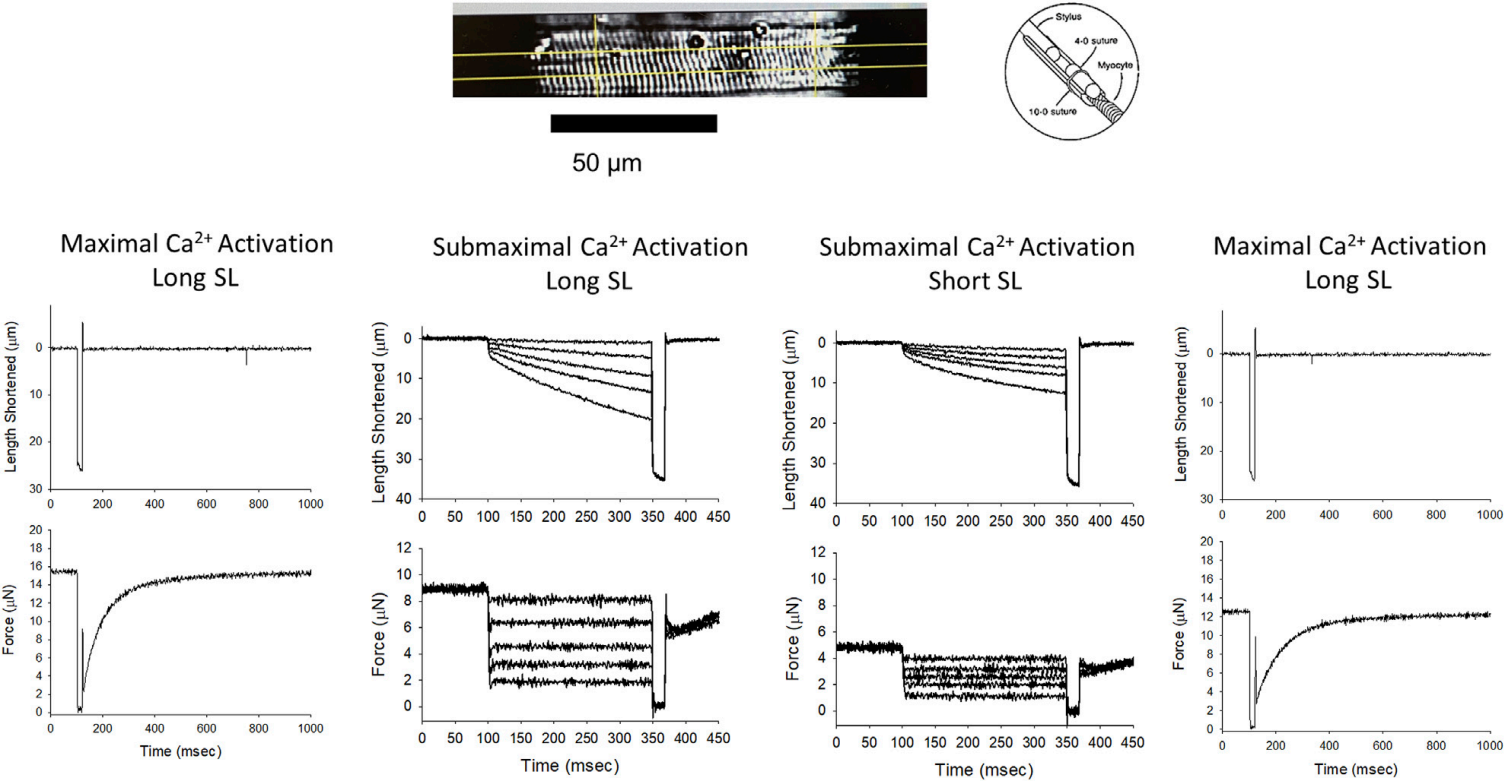
Permeabilized cardiac myocyte preparation and experimental workflow. *Top*, a photomicrograph of a representative mouse permeabilized cardiac myocyte preparation and a schematic showing the permeabilized myocyte attachment. The *bottom* panel shows length and force traces during maximal Ca^2+^ activation at the beginning of the experiment, followed by representative length traces during a series of force clamps at both long and short sarcomere lengths during sub-maximal Ca^2+^ activation, and a final length and force trace during maximal Ca^2+^ activation.

Contractile properties were measured in permeabilized cardiac myocyte-sized preparations from WT and Dmd^mdx-4cv^ mice. The characteristics of permeabilized cardiac myocyte preparations from both groups are provided in Table 1. Figure 2 illustrates experimental workflow for assessment of contractile properties of permeabilized cardiac myocyte preparations. First, force and rate of force development were measured during maximal Ca^2+^ activation. This was followed by measurements of force, rate of force, and loaded shortening during sub-maximal (∼half-maximal) Ca^2+^ activation at both long (∼2.25 μm) and then short (∼1.95 μm) sarcomere length (SL), followed by a final force measurement during maximal Ca^2+^ activation (Figure 2).

**TABLE 1:** Wildtype (WT) and Mdx-^4cv^ mouse permeabilized cardiac myocyte preparations at long sarcomere length (SL).

	Cardiac myocyte preparations	Length (µm)	Width (µm)	SL (µm)	Passive tension (kN·m^-2^)	Maximum force (µN)	Maximum tension (kN·m^-2^)	pCa for sub-max tension	Relative tension sub-max pCa
WT (N = 7)	n = 7	115 ± 9	21 ± 1	2.29 ± 0.01	2.07 ± 0.33	10.2 ± 1.2	46 ± 7	6.06 ± 0.02	0.56 ± 0.03
Mdx-^4cv^ (N = 8)	n = 10	123 ± 4	25 ± 1	2.28 ± 0.02	1.31 ± 0.22	13.6 ± 1.6	45 ± 7	6.09 ± 0.02	0.50 ± 0.03

Values are means ± SEM. N = number of mice (Biological replicates).

**FIGURE 3:**
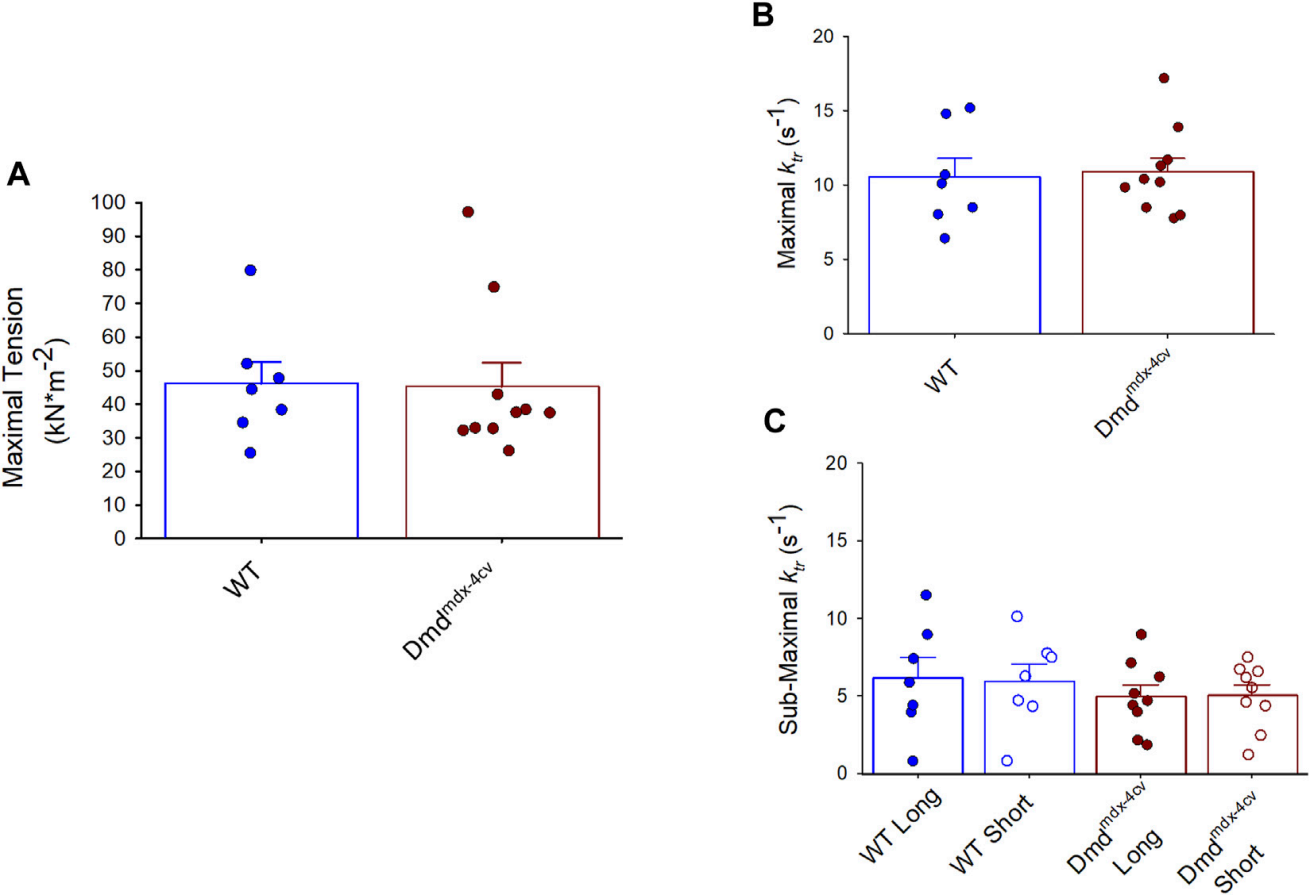
**(A)**. Maximal Tension and **(B)**. rates of force redevelopment (k_tr_) were similar in permeabilized cardiac myocyte preparations from either wildtype (WT) or Dmd^mdx-4cv^ mice. **(C)**. The sarcomere length dependence of force redevelopment (k_tr_) during sub-maximal Ca^2+^ activation also showed no differences between WT and Dmd^mdx-4cv^ permeabilized cardiac myocyte preparations. k_tr_ measurements were compared pairwise at long and short sarcomere length during sub-maximal Ca^2+^ activations.

Maximal tension was similar between WT and the Dmd^mdx-4cv^ permeabilized cardiac myocyte preparations (WT: 46 ± 17 kN × m^-2^; Dmd^mdx-4cv^ 45 ± 23 kN × m^-2^) (Figure 3A). Additionally, there was no difference in rates of force development (as indexed by the rate constant, *k*_*tr*_) following slack-restretch manoeuvre during maximal Ca^2+^ activations between WT and Dmd^mdx-4cv^ myocyte preparations (WT: 10.5 ± 1.3 s^-1^; Dmd^mdx-4cv^: 10.9 ± 0.9 s^-1^) (Figure 3B). Sub-maximal force development rates (k_tr_) were also similar between WT and Dmd^mdx-4cv^ preparations at long SL and short SL (WT: Long SL: 6.13 ± 3.53 s^-1^, Short SL: 5.94 ± 2.99 s^-1^, Dmd^mdx-4cv^: Long SL: 4.96 ± 2.27 s^-1^, Short SL: 5.02 ± 2.08 s^-1^) (Figure 3C).

**FIGURE 4:**
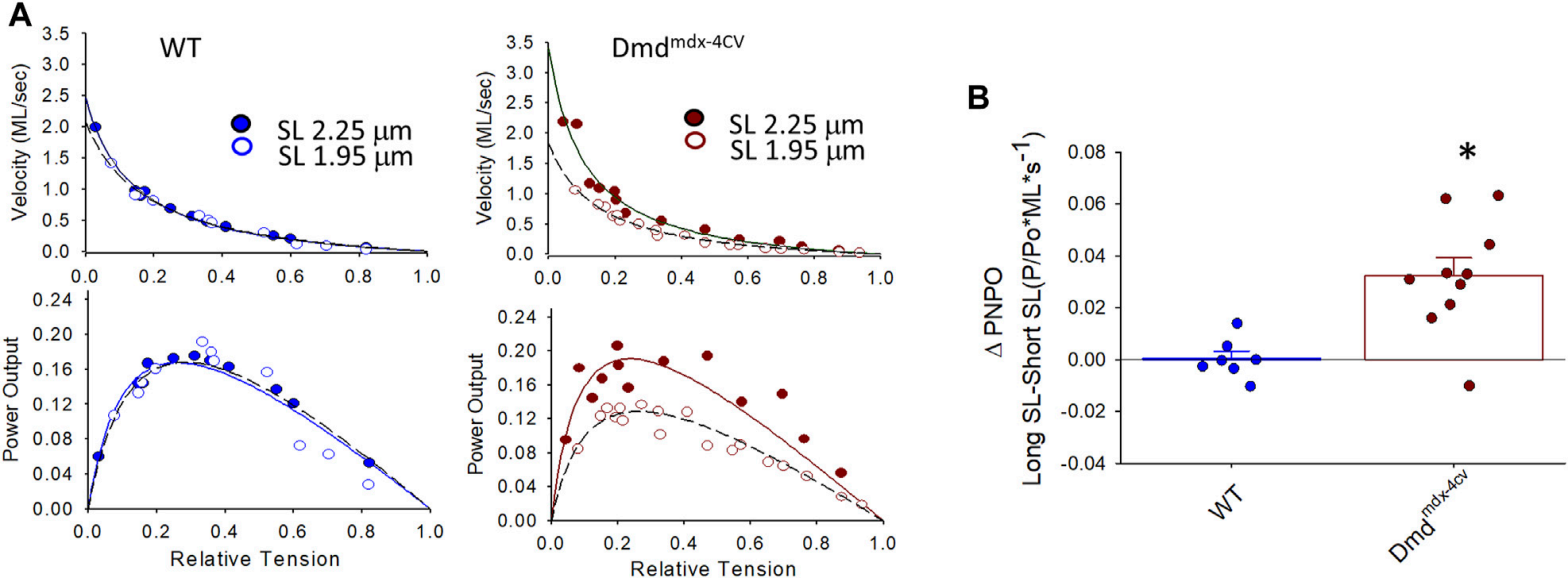
**(A)**. Representative normalized force-velocity and power-load curves at long sarcomere length (∼2.25 µm) and short sarcomere length (∼1.95 µm) in a permeabilized cardiac myocyte preparation from a wildtype (WT) (left) and a Dmd^mdx-4cv^ mouse (right). Units for peak normalized power output (PNPO) are (P/P_0_*ML*s^-1^) **(B)**. Change in peak normalized power output (ΔPNPO) between long sarcomere length and short sarcomere length in permeabilized cardiac myocyte preparations from WT littermates and Dmd^mdx-4cv^ mice. Length dependence of PNPO was significantly greater in permeabilized cardiac myocyte preparations from Dmd^mdx-4cv^ mice.

Figure 4 shows representative force-velocity and power-load relationships (normalized to isometric force) from a permeabilized cardiac myocyte preparation from a WT mouse heart (left panel) and a Dmd^mdx-4cv^ mouse heart (right panel). Table 2 shows force-velocity and power-load characteristics at long and short sarcomere lengths from permeabilized cardiac myocyte preparations between groups. Interestingly, there was a greater sarcomere length dependence of peak normalized power output in permeabilized cardiac myocyte preparations from Dmd^mdx-4cv^ mice (WT: ΔPNPO long SL-short SL: 0.0004 ± 0.0076, Dmd^mdx-4cv^: ΔPNPO long SL-short SL: 0.032 ± 0.022; *p* < 0.05) (Figure 4).

**TABLE 2:** Permeabilized cardiac myocyte preparation force-velocity and power-load characteristics at long and short sarcomere length (SL) during sub-maximal Ca^2+^ activation.

		SL (µm)	*F*_opt_ (*P/P*_*0*_)	*V*_*opt*_ (*ML·s* ^*-1*^)	*V*_max_ (*ML·s* ^*-1*^)	Peak absolute power output (µW mg^-1^)	Peak normalized power output (*P/P*_*0*_ *·ML·s* ^*-1*^)
WT	Long SL	2.29 ± 0.01	0.29 ± 0.02	0.53 ± 0.06	1.68 ± 0.17	3.85 ± 0.71	0.155 ± 0.022
	Short SL	1.95 ± 0.00	0.30 ± 0.02	0.52 ± 0.05	1.75 ± 0.19	2.39 ± 0.44	0.156 ± 0.020
Mdx^-4cv^	Long SL	2.28 ± 0.02	0.29 ± 0.02	0.58 ± 0.04	2.07 ± 0.21	3.32 ± 0.54	0.170 ± 0.014
	Short SL	1.95 ± 0.00	0.30 ± 0.02	0.48 ± 0.03	1.59 ± 0.11	1.51 ± 0.29	0.138 ± 0.012

Values are means ± SEM.

**FIGURE 5:**
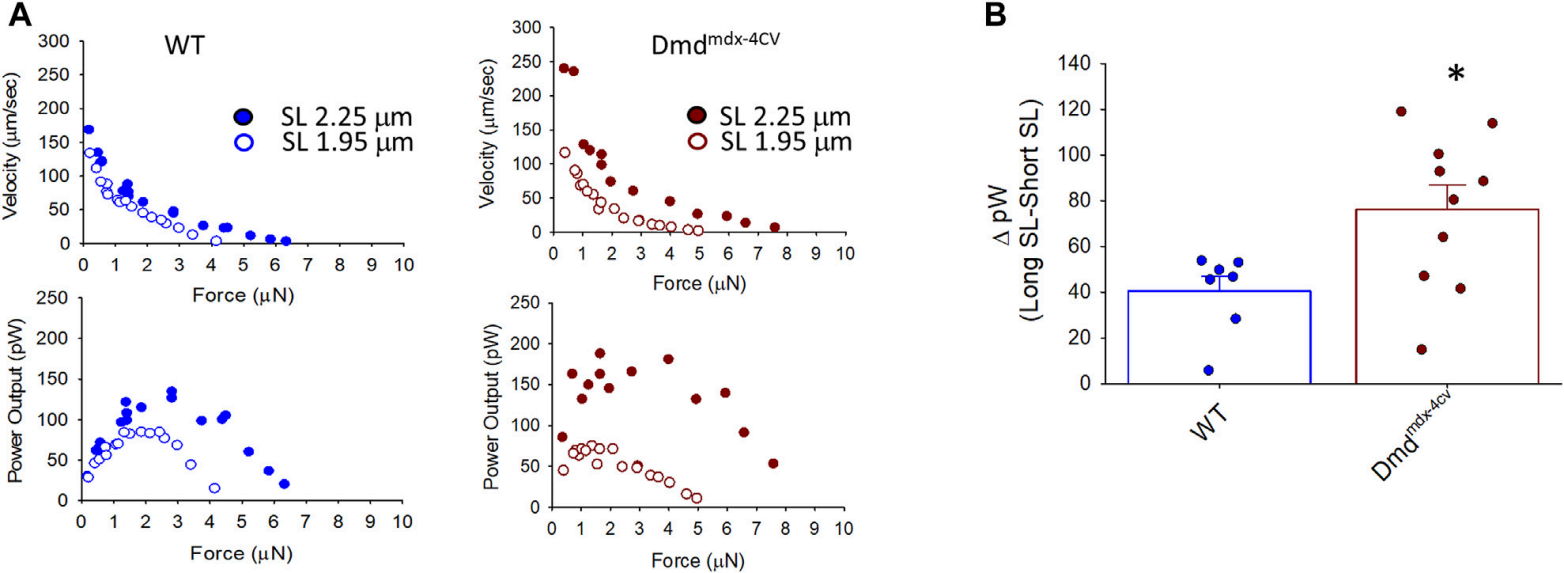
**(A)**. Representative absolute force-velocity and peak power-load curves at long sarcomere length (∼2.25 µm) and short sarcomere length (∼1.95 µm) in a permeabilized cardiac myocyte preparation from a wild-type (WT) (left) and a Dmd^mdx-4cv^ mouse (right). **(B)**. Change in peak absolute power (ΔpW) from long sarcomere length to short sarcomere length in cardiac myocyte preparations from WT littermates and Dmd^mdx-4cv^ mice. Length dependence of peak absolute power was significantly greater in permeabilized cardiac myocyte preparations from Dmd^mdx-4cv^ mice.

Length dependence of absolute power was also determined to look at the effects of dystrophin-deficiency on the product of force and loaded shortening. Figure 5 shows sarcomere length dependence of peak absolute power of cardiac myocyte preparations between groups. Length dependence of peak power (pW) was greater in the Dmd^mdx-4cv^ preparations compared to the WT preparations. From short SL to long SL power output increased 85.67 ± 40.58 pW in the Dmd^mdx-4cv^ preparations but only 31.00 ± 19.52 pW in the WT preparations. The sarcomere length dependence of tension was similar between groups (see Figure 8). These results suggest the loss of dystrophin increases sarcomere length dependence of loaded shortening and peak power in Dmd^mdx-4cv^ cardiac myofilaments.

### Mavacamten treatment

Since we found greater sarcomere length dependence of loaded shortening and power in dystrophic cardiac myocytes, we next investigated the effects of mavacamten on contractile properties of cardiac myofilaments from DMD mice. Mavacamten has been shown to decrease contractility in myocardial preparations (Green et al., 2016; Awinda et al., 2021), making it a promising drug to treat hypercontractility disorders such as HCM and, it stands to reason, that it may be beneficial for dystrophic cardiomyopathy, which is thought to arise, at least in part, from contraction-induced damage. Mavacamten (0.5 µM) was added to pCa solutions and contractile properties were measured in permeabilized cardiac myocytes from WT and Dmd^mdx-4cv^ mice. The characteristics of permeabilized cardiac myocyte preparations from both treatment groups are provided in Table 3.

**TABLE 3:** Mavacamten- wildtype (WT) and Mdx-^4cv^ mouse permeabilized cardiac myocyte preparations at long sarcomere length (SL).

	Cardiac myocyte preparations	Length (µm)	Width (µm)	SL (µm)	Passive tension (kN·m^-2^)	Maximum force (µN)	Maximum tension (kN·m^-2^)	Maximum tension with mava (kN·m^-2^)	pCa for sub-max tension	Relative tension sub-max pCa
WT (N = 3)	n = 3	115 ± 15	20 ± 2	2.31 ± 0.01	1.42 ± 0.52	11.9 ± 1.2	53 ± 8	29 ± 5	5.7 ± 0.1	0.66 ± 0.05
Mdx-^4cv^ (N = 4)	n = 4	147 ± 21	29 ± 3	2.28 ± 0.03	1.24 ± 0.56	22.8 ± 5.3	48 ± 6	26 ± 4	5.7 ± 0.1	0.54 ± 0.04

Values are means ± SEM. N = number of mice (Biological replicates).

**FIGURE 6:**
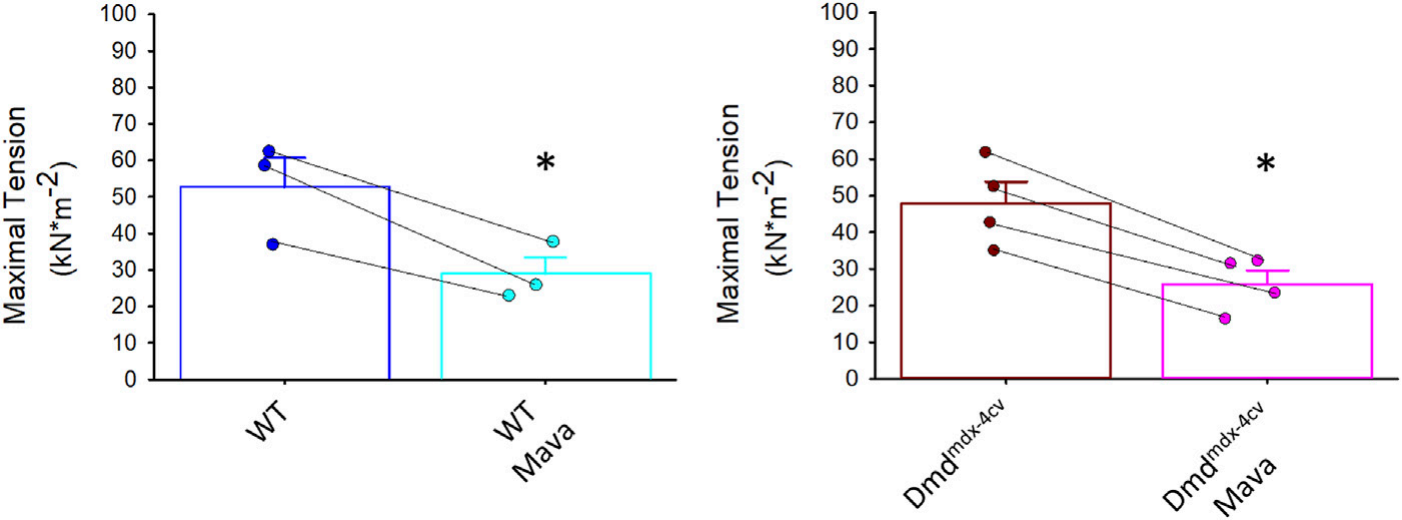
Maximal tension in absence and presence of mavacamten (Mava) in permeabilized cardiac myocyte preparations from wildtype (WT) and Dmd^mdx-4cv^ mice. Maximal tension was significantly decreased in both WT and Dmd^mdx-4cv^ myocytes with mavacamten.

Figure 6 shows that mavacamten significantly decreased maximal tension (by ∼45%) in cardiac myocyte preparations from both WT and Dmd^mdx-4cv^ mice. This result is consistent with the tension decline by mavacamten previously reported in permeabilized multi-cellular myocardial preparations (Awinda et al., 2021). These results indicate that mavacamten mitigates tension generation in permeabilized cardiomyocytes regardless of dystrophin content.

**FIGURE 7:**
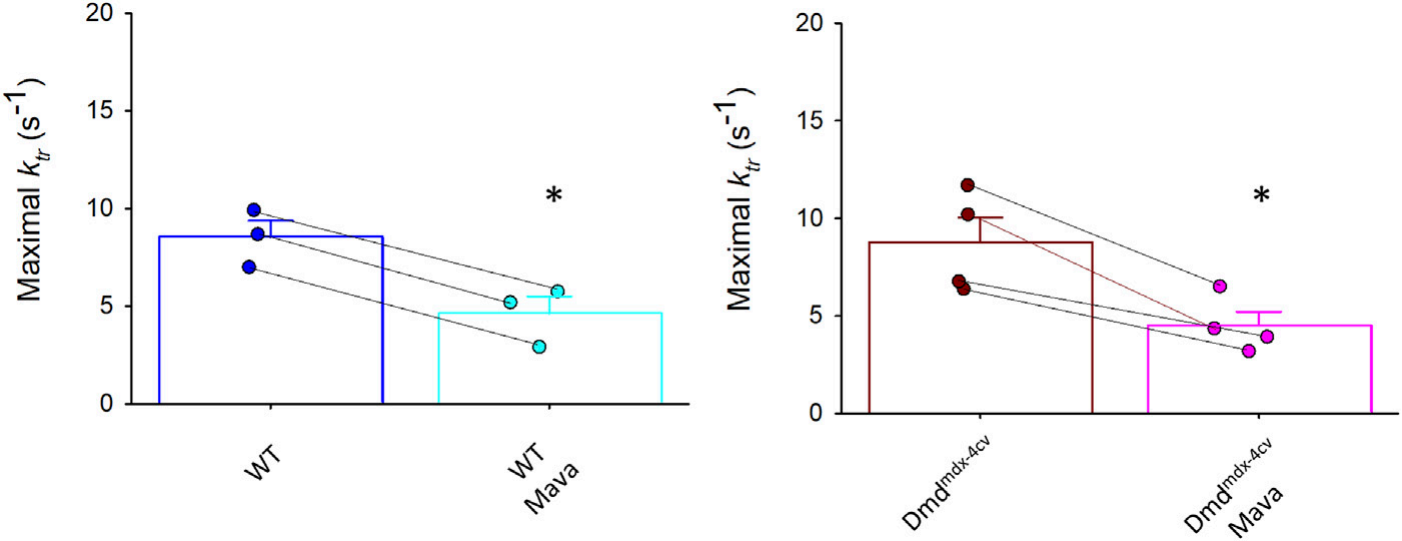
Rates of force redevelopment (k_tr_) during maximal Ca^2+^ activation were significantly decreased in both wildtype (WT) and Dmd^mdx-4cv^ myocytes in presence of mavacamten (Mava).

**FIGURE 8:**
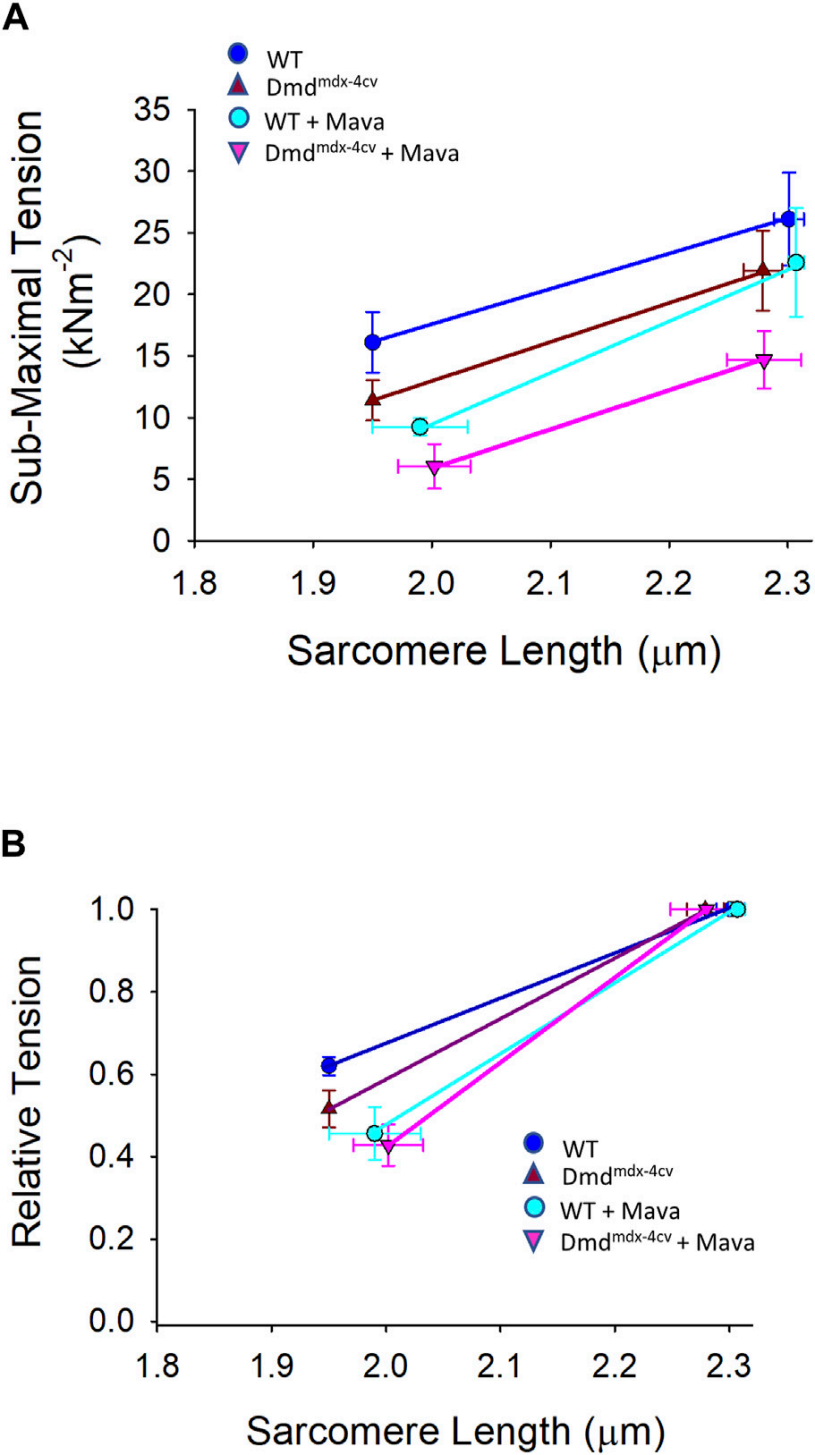
**(A)**. Absolute and **(B)**. relative sarcomere length-tension relationships for wildtype (WT) and Dmd^mdx-4cv^ myocytes in the absence and presence of mavacamten (Mava) treatment.

Maximal Ca^2+^ activated rates of force development (*k*_*tr*_ values) were also significantly decreased by mavacamten in both WT and Dmd^mdx-4cv^ preparations, highlighting the ability of mavacamten to decrease force kinetics in single permeabilized cardiac myocyte preparations (Figure 7). Intriguingly, mavacamten tended to steepen absolute and normalized sarcomere length-tension relationships in cardiac myocyte preparations from both WT and Dmd^mdx-4cv^ preparations (Figure 8B), similar to a previous report (Sewanan et al., 2021).

**FIGURE 9:**
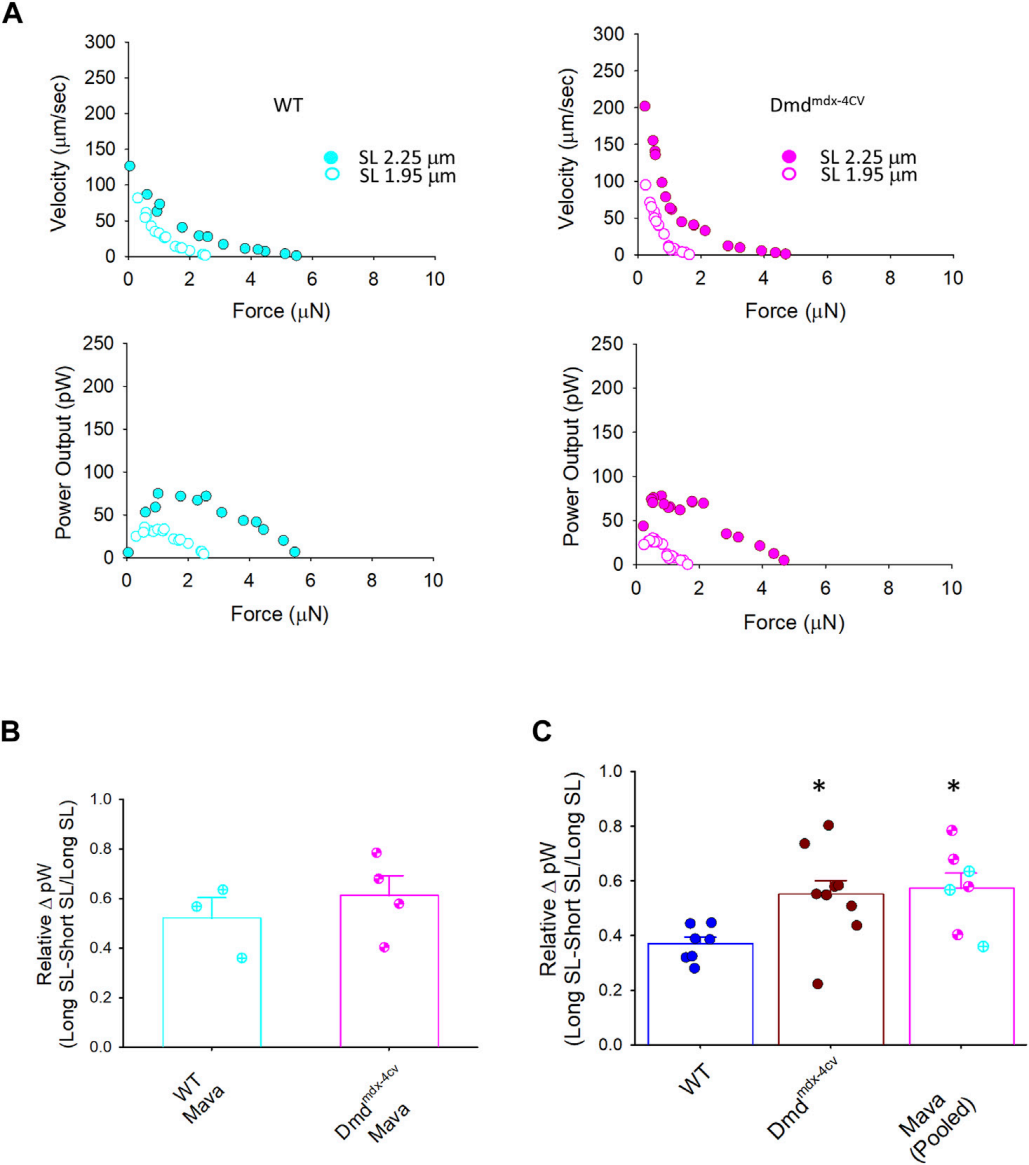
Change in peak power (expressed as relative ΔpW) between WT, Dmd^mdx-4cv^, and mavacamten (Mava)-treated permeabilized cardiac myocyte preparations. **(A)**. Representative absolute force-velocity and power-load relationships from permeabilized cardiac myocyte preparations from a WT mouse heart (left panel) and a Dmd^mdx-4cv^ mouse heart (right panel). **(B)**. Mavacamten resulted in a similar length dependence of peak power (relative pW) between WT and Dmd^mdx-4cv^ permeabilized cardiac myocyte preparations. **(C)**. Mavacamten normalized the difference in length dependence of peak power previously observed between WT and Dmd^mdx-4cv^ permeabilized cardiac myocytes. For the Mava group, WT (*cyan* circles crossed) and Dmd^mdx-4cv^ (*pink* circles hourglass) myocyte preparations were pooled since there was no difference between the two groups in panel **(B)**.

Table 4 shows force-velocity and power-load characteristics at long and short sarcomere lengths from permeabilized cardiac myocyte preparations treated with mavacamten. Figure 9A shows representative absolute force-velocity and power-load relationships from a permeabilized cardiac myocyte preparation from a WT mouse heart (left panel) and a Dmd^mdx-4cv^ mouse heart (right panel). Interestingly, mavacamten eliminated any difference in sarcomere length dependence of power in cardiac myocyte preparations between WT and Dmd^mdx-4cv^ groups (Figure 9B). Sarcomere length dependence of absolute peak power (expressed in relative terms) shifted to a higher value in the WT myocyte preparations after mavacamten (Figures 9B,C). In fact, sarcomere length dependence of power with mavacamtem was similar to Dmd^mdx-4cv^ myocyte preparations without mavacamten (Figure 9C). These results implicate that, while mavacamten decreases overall contractility, it maintains or even augments sarcomere length dependence of contraction in cardiac myofilaments.

**TABLE 4:** Mavacamten- permeabilized cardiac myocyte preparation force-velocity and power-load characteristics at long and short sarcomere length (SL) during sub-maximal Ca^2+^ activation.

		SL (µm)	*F*_opt_ (*P/P*_*0*_)	*V*_*opt*_ (*ML·s* ^*-1*^)	*V*_max_ (*ML·s* ^*-1*^)	Peak absolute power output (µW mg^-1^)	Peak normalized power output (*P/P*_*0*_ *·ML·s* ^*-1*^)
WT	Long SL	2.31 ± 0.01	0.29 ± 0.02	0.37 ± 0.07	1.27 ± 0.18	2.80 ± 1.32	0.111 ± 0.025
	Short SL	1.99 ± 0.04	0.28 ± 0.02	0.39 ± 0.06	1.42 ± 0.20	1.16 ± 0.41	0.109 ± 0.022
Mdx^-4cv^	Long SL	2.28 ± 0.03	0.26 ± 0.02	0.52 ± 0.07	2.03 ± 0.28	2.01 ± 0.59	0.132 ± 0.019
	Short SL	2.00 ± 0.03	0.27 ± 0.02	0.40 ± 0.08	1.60 ± 0.29	0.76 ± 0.29	0.103 ± 0.019

Values are means ± SEM.

## Discussion

Duchenne Muscular Dystrophy (DMD) is a devastating form of dystrophinopathy. DMD is an X-linked recessive disease resulting from a complete absence of the dystrophin protein. Advances in treatment of skeletal muscle, such as corticosteroid therapy, have revealed a previously unappreciated cardiac component of the disease (Matthews et al., 2016; Cheeran et al., 2017; Shih et al., 2020). As such the prevalence of DMD patients with heart failure is increasing, underscoring the importance of mechanistic studies for rationale treatment strategies. While some previous studies have examined the effect of DMD on contractile properties, there are no reports, to our knowledge, investigating the effect of DMD on power generating capacity of single, permeabilized cardiac myocyte preparations.

The focus of this study was to examine how the loss of dystrophin affects contractile properties of permeabilized cardiac myocyte preparations in a mouse model of DMD. Contractility measurements were performed on permeabilized cardiac myocyte preparations from WT and Dmd^mdx-4cv^ mice. Maximal Ca^2+^ activated tension was similar between preparations from WT and Dmd^mdx-4cv^ groups. This differs slightly from other studies; for instance, Janssen et al. (Janssen et al., 2005; Xu et al., 2011) reported decreased twitch force in isolated trabeculae from mdx mice. This difference may reflect alterations in excitation-contraction coupling in the absence of dystrophin. Also, Su et al. (Su et al., 2012) reported small reductions in maximal Ca^2+^ activated tension in permeabilized myocyte preparations from sub-endocardium of Golden Retriever Muscular Dystrophy (GRMD) left ventricles. Overall, the varied results may arise from differences in species, mouse strains, age, disease severity, and muscle preparations.

We also found no difference in maximal rates of force development between preparations from WT and Dmd^mdx-4cv^ groups, which is like a previous study using permeabilized myocyte preparations from GRMD hearts (Ait Mou et al., 2018). However, we did observe a greater sarcomere length dependence of power output in myocyte preparations from Dmd^mdx-4cv^ mice. The increased sarcomere length dependence of power could potentially exacerbate hemodynamic load-dependent myocyte damage in DMD hearts (Danialou et al., 2001). Accordingly, this potentially elucidates a mechano-transduction mechanism by which myocytes lacking dystrophin have a greater propensity for ventricular damage and consequent cardiomyopathy leading to failure.

The greater sarcomere length dependence of power in Dmd^mdx-4cv^ permeabilized cardiac myocytes led us to test whether the myosin cross-bridge stabilizing peptide, mavacamten, might mitigate this response. As expected, mavacamten decreased maximal tension, maximal *k*_*tr*_, and power in both wild-type and Dmd^mdx-4cv^ cardiac myocyte preparations. Mavacamten also normalized sarcomere length dependence of power between groups. Interestingly though, mavacamten, instead of decreasing delta power in the Dmd^mdx-4cv^ cardiac myocytes, increased delta power in WT myocytes (Figure 9B) values that matched DMD^mdx-4cv^ levels.

These findings show promise for mavacamten as a potential treatment for DMD. Mavacamten decreased both maximal tension and force development kinetics. Since patients with DMD are more susceptible to contraction-induced damage, attenuation of force and rate of force by mavacamten may offer a new avenue to lessen damage induced by myocyte stress. While we predicted that mavacamten may minimize sarcomere length dependence of contractile properties, this was not the case; in fact, mavacamten appeared to augment sarcomere length dependence of contractile properties. Interestingly, while conventional negative iontropes (e.g., beta-blockers) also decrease contractility, they tend to diminish sarcomere length dependence of power (Hanft and McDonald, 2010) and attenuate Ca^2+^ cycling kinetics (Solaro, 2002). Thus, mavacamten may be better suited to treat contraction-induced damage disorders by its direct action to reduce force and kinetics and overall cardiac work (Danialou et al., 2001) yet still maintaining exquisite sarcomere length dependence of contractility, i.e., the ventricles retain their intrinsic capacity to rapidly respond to changes in metabolic demand by the Frank-Starling mechanism (i.e., greater end diastolic volumes elicit proportional increases in stroke volume). Testing this hypothesis will require systematic delineation of how mavacamten modulates function across different levels of myocardial organization in DMD-associated cardiomyopathy.

## Data Availability

The raw data supporting the conclusion of this article will be made available by the authors, without undue reservation.
